# Person-centered maternity care during childbirth and associated factors at selected public hospitals in Addis Ababa, Ethiopia, 2021: a cross-sectional study

**DOI:** 10.1186/s12978-022-01503-w

**Published:** 2022-10-04

**Authors:** Azezew Ambachew Tarekegne, Berhanu Wordofa Giru, Bazie Mekonnen

**Affiliations:** 1 St. Paul’s Hospital Millennium Medical College, Addis Ababa, Ethiopia; 2 College of Health Sciences, School of Nursing and Midwifery, Addis Ababa University, Addis Ababa, Ethiopia

**Keywords:** Addis Ababa, Childbirth, Ethiopia, Person-centered maternity care

## Abstract

**Background:**

Person-centered maternity care is respectful and responsive care to individual women’s preferences, needs, and values and ensuring that their values guide all clinical decisions during childbirth. It is recognized as a key dimension of the quality of maternity care that increases client satisfaction and institutional delivery. However, little research has been conducted about person-centered maternity care in Ethiopia.

**Objective:**

The aim of this study was to assess the status of person-centered maternity care and associated factors among mothers who gave birth at selected public hospitals in Addis Ababa city, Addis Ababa, Ethiopia, 2021.

**Method:**

A facility-based cross-sectional study was conducted at selected public hospitals in Addis Ababa city. A structured questionnaire was used to collect data from post-natal mothers selected by systematic random sampling. Data were collected using face-to-face interview technique. The data was coded and entered using Epi-data version 4.6 and analyzed using SPSS version 25. Bivariate and multivariable linear regression analysis was used to identify factors associated with person-centered maternity care. The strength of association between independent and dependent variables was reported by using unstandardized β at 95% CI and p-value < 0.05 were considered statistically significant.

**Results:**

In this study 384 mothers were participated with a response rate of 99.2%. The overall prevalence of person-centered maternity care was 65.8% and the percentage mean Person Centered Maternity Care (PCMC) score of the respondents was 65.8% with percentage standard deviation of 17.06. Respondents who had no ANC follow-up (β = -5.39, 95% CI: -10.52, -0.26), < 4 Antenatal Care (ANC) follow up (β = -3.99, 95% CI: -6.63, -1.36), night time delivery (β = -3.95, 95% CI: -5.91, -1.98) and complications during delivery (β = -3.18, 95% CI: -6.01, -0.35) were factors significantly associated with person-centered maternity care.

**Conclusion and Recommendations:**

The finding of this study showed that the proportion of person-centered maternity care among mothers who gave birth in public hospitals of Addis Ababa was high as compared to previous studies. The factors affecting person-centered maternity care are manageable to interventions. Therefore, Policymakers should develop and implement guidelines about person-centered maternity care. Training should be given to health care providers on the importance of person-centered maternity care and patient and provider rights. Hospital managers should increase the number of staffs who got PCMC training, especially during nighttime to improve the provision of person-centered maternity care. Health care providers should implement person-centered maternity care for all mothers who gave birth in the health care facility.

## Plain language summary

Person-centered maternity care is a key dimension of quality of maternity care that can increase client satisfaction and affects health-seeking behavior of the community. PCMC approach is believed to advance communication between women and health care providers, making care empathetic and improve maternal and neonatal health outcomes.. 384 mothers who gave birth in selected public health hospital were enrolled with an overall prevalence of 65.8%. Respondents who had no antenatal care (ANC) follow-up, < 4 ANC follow up, night time delivery, and complications during delivery were factors that were found to be associated with person-centered maternity care.

Health care providers, Health institutions, Policy makers, and Health sector officials should design and implement training strategies to provide highlighted dignity and respect, communication and autonomy, and supportive care for all mothers during labor and delivery.

## Introduction

### Background

Person-centered maternity care (PCMC) is defined as providing maternity care that’s responsive and respectful to individual women’s preferences, values, and needs; ensuring that women’s values guide all clinical decisions during labor and childbirth [1, 2]. The World Health Organization (WHO) recommendations highlighted dignity and respect, communication and autonomy, and supportive care during childbirth as key components of PCMC that should be provided for all mothers during labor and delivery. It aims to improve communication between health care providers and women to promote the utilization of care [3, 4].

Person-centered care during childbirth is identified as a key dimension of the quality of maternity carethat recognizes user experience and affects health-seeking behavior [3]. Studies showed that the PCMC approach can lead to decreased maternal and neonatal complications, postpartum depression, and improved patient satisfaction. Moreover, when women feel respected and experience compassion from care providers during labor and delivery they are more likely to return for postpartum maternal health services [5–7].

Poor quality of care contributes to high maternal mortality in developing countries, especially in Sub-Saharan. About three-quarters of maternal deaths are due to complications of labor, childbirth, and the first 24 h postpartum after delivery [8, 9].Evidence showed that person-centered maternity care plays an important role in the identification of complications during facility-based childbirth thus reducing maternal mortality and morbidity significantly. Moreover, PCMC emphasizes the quality of patient experience by helping the woman to feel safe and at ease to communicate how she feels and what she needs to the health care provider [5, 10].

Poor person-centered maternity care which is characterized by the disrespectful and abusive treatment of women during facility-based childbirth can deter women from giving birth in health facilities and lead to poor maternal and neonatal outcomes [11–14]. It has psychological effects for mothers, a higher risk of dissolution and risk for families, and the potential poverty of thousands due to high costs of care [15, 16].

Advancing PCMC approaches in maternal health services is essential to improve client satisfaction, increase facility-based deliveries and ensure effective implementation of women’s rights [6, 15]. Efforts to increase maternal utilization of health services in low and middle-income countries are not possible to achieve the desired goals without improving women’s experience of care. Other studies showed that women intent to give birth in an environment where they feel safe, valued, and respected [4, 12, 17].

A study in low and middle-income countries revealed that mothers were not experiencing person-centered maternity care. This study reported that women were receiving the highest mean PCMC score (66.9%) in urban Kenya and the lowest PCMC score (51.6%) in rural Ghana. The study reported that health care providers never introduced themselves for 90% of mothers, 53% of women in Kenya, and 73% of women in India were not asked permission from health care providers before doing procedures [18].

Shreds of evidence showed that person-centered maternity care was affected by the socioeconomic status of the women and by health facility level [10, 18, 19]. A study done in Kenya found that wealthier, employed, literate and married women experienced higher person-centered maternity care scores as compared to poorer, unemployed, illiterate, and unmarried women respectively [8].

A study done in Ethiopia reported that 64.5% of women experienced person-centered maternity care. This study also showed that person-centered maternity care increases client satisfaction and influences the health-seeking behavior of women [2]. On the other hand, poor person-centered maternity care results in decreased institutional delivery [20, 21]. Residence, average monthly income, having antenatal care (ANC) follow-up, time of delivery, mode of delivery, complication during childbirth, and length of stay in the health facility were factors affecting person-centered maternity care [2, 22–25].

Several studies have been conducted to identify determinant factors of person-centered maternity care in different countries. However, little research has been conducted in Ethiopia about person-centered maternity care during facility-based childbirth. Therefore this study aimed to assess the status of person-centered maternity care during childbirth and associated factors at public hospitals in Addis Ababa, Ethiopia.

## Methods

### Study area, period, and design

A facility-based cross-sectional study was conducted in public hospitals of Addis Ababa city from February 15 to March 14/2021. Addis Ababa is the capital city of Ethiopia and covers an area of 527 km. There were 13governmental hospitals in the city. From those, five hospitals were governed by the Federal Ministry of Health, six governed by Addis Ababa health bureau, one under the police force, and one governed by the armed force, twelve hospitals provided labor and delivery services. The study was conducted in Tikur Anbessa Specialized Hospital, St. Paul’s Hospital Millennium Medical College, Gandhi Memorial Hospital, and Yekatit 12 Hospital.

Tikur Anbessa Specialized Hospital is a referral and teaching hospital under the Ministry of Education of Ethiopia. St. Paul’s Hospital Millennium Medical College is a specialized and teaching hospital managed under the Federal Ministry of Health. Gandhi Memorial Hospital and Yekatit 12 Hospital are governmental hospitals administered under the Addis Ababa health bureau.

### Population, sample size determination and sampling technique

The source population was all women who gave birth at selected public hospitals in Addis Ababa and the study population was randomly selected women (aged above 18 years) who gave birth in selected hospitals of Addis Ababa during the data collection period.

The sample size was determined by using a single population proportion formula. A previous study conducted to assess the determinants of person-centered maternity care in Dessie town, Ethiopia reported the prevalence of person-centered maternity care was 64.5% [2]. This study used the assumption of standard normal distribution at a 95% confidence level and a margin of error assumed to be 5%. Considering a non-response rate of 10%, the final sample size was 387.

Four hospitals were selected out of thirteen governmental hospitals in Addis Ababa by simple random sampling. Therefore, Tikur Anbessa Specialized Hospital, Gandhi Memorial Hospital, St. Paul’s Hospital Millennium Medical College, and Yekatit 12 Hospital were the required hospitals for this study. The total sample size (387) was allocated proportionally to each of the selected hospitals by reviewing the number of deliveries attended in each hospital. A systematic random sampling technique was used to collect data using the women’s delivery registration logbook during the study period in each hospital using face-to-face interview technique. K = N/n = 2050/387 = 5. N = 2050 was the sum total of mothers attending those selected public hospitals for maternity care from whom the study population was selected. Before applying kth value, the first mother was selected from the first five women using lottery method. After that every fifth mother was interviewed.

### Study variables

The dependent variable was person-centered maternity care and the independent variables included socio-demographic characteristics (age, marital status, residence, level of education, employment status, and monthly income), obstetrics related factors (parity, antenatal care (ANC), mode of delivery, time of delivery, previous delivery experience, complication during delivery, newborn outcome, sex of delivery attendant, and profession of delivery provider) and facility characteristics such as availability of infrastructure and length of stay at a health facility.

### Operational definitions

#### Person-centered maternity care

Person-centered maternity care was measured by using the PCMC scale. The PCMC scale has three domains: dignity and respect, communication and autonomy, and supportive care and 30 items with each item having a four-point response scale, that is 0- ‘no, never’, 1- ‘yes, a few times, 2- ‘yes, most of the time’ and 3- ‘yes, all the time’. The negative items such as physical abuse, verbal abuse, auditory privacy, and crowdedness of the room questions were reversely coded so that the highest score represents good care. The total PCMC score is a summative score from the response to individual items which ranges from 0 to 90 [2, 10]^.^

#### Dignity and respect

It was measured by using six items with each item having a four-point response scale.

#### Communication and autonomy

It was measured by using nine items with each item having a four-point response scale.

#### Supportive care

It was measured by using fifteen items with each item having a four-point scale.

#### Data collection tool and procedures

Structured questionnaires which have socio-demographic characteristics of the mother, obstetrics history, and person-centered maternity care scale were used to collect the data from study participants. The tool was validated in different low and middle-income countries including Ethiopia to assess person-centered care for developing settings. The scale has good internal reliability with Cronbach’s alpha above 0.8 [2, 8, 26].

The data was collected from mothers who gave birth in selected public health hospitals using a standardized, pretested, and structured Amharic version questionnaire. Four Diploma Midwives were recruited for data collection and two Bachelors of sciences Midwives were assigned for supervision.

### Data quality assurance

The questionnaire for data collection was prepared first in English and translated into Amharic and back-translated to English to keep the consistency of the data. One-day training was given for data collectors and supervisors by investigators on the roles and responsibilities as well as overall data collection processes, and on accuracy and reliability of data gathering procedures. Additionally, a pre-test of the questionnaire was done on 5% of the sample size (20 women) who gave birth in Zewditu Memorial Hospital in Addis Ababa City. The data was checked daily for its completeness and consistency by the investigator and supervisors.

### Data processing and analysis

The data were checked for completeness, cleaned manually, coded and entered into Epi-data version 4.6 software, and exported to SPSS version 25 for further analysis. Descriptive statistics were presented using tables and figures.

After creating dummy variables simple linear regression analysis was used primarily to check which variables had an association with the dependent variable. The assumption of linearity was checked by plotting a P-P plot and normality was checked by using Q-Q plots and histogram. Multi co-linearity assumption was checked by Variance Inflation Factor (VIF) and there is no multi co-linearity. Variables having p-value ≤ 0.25 in simple linear regression were entered into multiple linear regressions for controlling the possible effect of confounders by enters method. Factors that had significant association were identified by using unstandardized β, 95% confidence interval (CI), and p-value ≤ 0.05.

## Results

### Socio-demographic characteristics of the respondents

In this study, 384 mothers participated with a response rate of 99.2%. Most of the study participants 328(85.4%) were from Addis Ababa. The mean age of the mothers was 26 (SD, ± 3.87) years with a minimum and maximum age of 18 and 40 years respectively.

Almost all of the respondents 369 (96.1%) were married. Out of the total respondents, 165 (43%) had a primary level of education. Regarding occupation, 226 (58.8) were housewives. The mean monthly income of the respondents was 3384 birr (Table 1).

**Table 1:** Socio demographic characteristics of the respondents at public hospitals of Addis Ababa, Ethiopia, 2021 (n = 384)

Variables	Frequency	Percentage (%)
Residence		
Urban	328	85.4
Rural	56	14.6
Age of mothers		
≤ 24	149	38.8
25–29	160	41.7
≥ 30	75	19.5
Marital status		
Married	369	96.1
Unmarried	15	3.9
Level of education		
Unable to read and write	23	6
Primary school (grade 1–8)	165	43
Secondary school (grade 9–12)	118	30.7
Diploma and above	78	20.3
Occupation		
Housewife	226	58.8
Government employee	34	8.9
Private employee	124	32.3
Income		
≤ 1650	62	16.1
1651–3200	174	45.3
> 3200	148	38.6

### Obstetrics characteristics of the respondents

From the total 384 study participants, almost all of 369 (96.1%) had antenatal care (ANC) follow-up for current delivery. Besides, nearly a quarter of the respondents 274 (74.3%) had received four and above ANC visits.

About 231 (60.2%) of the mothers were multiparous. Almost half of the mothers 188 (49%) had delivered through spontaneous vaginal delivery and 261 (68%) of deliveries were attended by Doctors. Over half of mothers 211 (54.9%) had given birth in the daytime. Most of the respondents 329 (85.7%) had no complications during delivery. Among the total respondents, 148 (36.8%) had more than two days of hospital stay (Table 2).

**Table 2:** Obstetrics characteristics of the respondents at public hospitals of Addis Ababa, Ethiopia, 2021 (n = 384)

Variables	Frequency	Percentage (%)
ANC		
Yes	369	96.1
No	15	3.9
Number of ANC		
< 4	95	25.7
≥ 4	274	74.3
Parity		
Primiparous	153	39.8
Multiparous	231	60.2
Number of facility based delivery		
1	156	40.6
≥ 2	228	59.4
Mode of delivery		
Spontaneous vaginal delivery	188	49
Cesarean section	176	45.8
Instrumental	20	5.2
The profession of delivery attendant		
Obstetricians and medical interns	261	68
Midwife	123	32
Sex of provider conducted delivery		
Male	248	64.6
Female	116	30.2
Both	20	5.2
Time of delivery		
Day	211	54.9
Night	173	45.1
Complication during delivery		
Yes	55	14.3
No	329	85.7
Newborn outcome		
Alive	368	95.8
Died	16	4.2
Length of stay in the hospital		
One day	186	48.4
Two days	50	13
More than two days	148	38.6

### Person-centered maternity care (PCMC) scales and sub-scales

The mean person-centered maternity care score of the respondents was 59.2 (SD =  ± 10.1) out of 90 with the minimum and maximum PCMC scores of 33 and 82 respectively. The percentage mean PCMC score of the respondents was 65.8%.

### Dignity and respect

The mean dignity and respect score of the respondents was 15.7 (SD =  ± 2.15) out of 18. About 133 (34.6%) of the study participants were treated with respect and 128 (33.3%) of participants were treated in a friendly manner during their stay in the facility. Only 15 (3.9%) and 4 (1%) of mothers had experienced verbal or physical abuse at least a few times in the facility respectively. In addition, 7 (1.8%) had reported their auditory privacy was not kept and 9 (2.3%) felt their health information was not kept confidential (Table 3).

**Table 3:** Distribution of dignity and respect items in public hospitals of Addis Ababa, Ethiopia, 2021(n = 384)

Items	No, never (%)	Yes, a few times (%)	Yes, most of the time (%)	Yes, all the time (%)
Providers treat me with respect	5 (1.3)	41 (10.7)	205 (53.4)	133 (34.6)
Provider treat me in a friendly manner	12 (3.1)	39 (10.2)	205 (53.4)	128 (33.3)
Providers shouted, insulted, scolded, threatened or talked me rudely (RC)	360 (93.7)	15 (3.9)	8 (2.1)	1 (0.3)
Providers pushed, slapped, beaten, pinched or physically restrained (RC)	378 (98.5)	4 (1)	2 (0.5)	0
People not involved in the care hear the discussion with the provider (RC)	354 (92.2)	7 (1.8)	19 (5)	4 (1)
Feel health information was or will be kept confidential	17 (4.4)	9 (2.3)	91 (23.7)	267 (69.6)

*RC* Reverse Coded

### Communication and autonomy

The mean communication and autonomy score of the respondents was 14.62 (SD =  ± 4) from 27. Most of the respondents 316 (82.3%) had reported providers never introduced themselves. Of the total respondents, 124 (32.3%) of mothers reported that providers never called by their name. Only 88 (22.9%) of respondents had been involved in decisions about their care during their stay in the facility. In addition 24 (6.3%) of the respondents reported that providers never asked consent before doing examinations and procedures.

Among the study participants, 233 (60.7%) of mothers were not allowed to be in a position of choice during delivery. Only 96 (25%) of the respondents reported that the purpose of medicines they took was explained and 67 (17.4%) of respondents reported that they could ask any questions they had to health care providers (Table 4).

**Table 4:** Distribution of communication and autonomy items in public hospitals of Addis Ababa, Ethiopia, 2021(n = 384)

Items	No, never (%)	Yes, a few times (%)	Yes, most of the time (%)	Yes, all the time (%)
Providers introduced themselves	316 (82.3)	45 (11.7)	21 (5.5)	2 (0.5)
Providers call me by my name	124 (32.3)	95 (24.7)	117 (30.5)	48 (12.5)
Involved in decisions	20 (5.2)	55 (14.3)	221 (57.6)	88 (22.9)
Consent before doing examinations and procedures	24 (6.3)	50 (13)	215 (56)	95 (24.7)
Allowed position of choice	233 (60.7)	83 (21.6)	62 (16.1)	6 (1.6)
Providers speak in a language that I could understand	5 (1.3)	12 (3.1)	104 (27.1)	263 (68.5)
The Purpose of examinations and procedures were explained	15 (3.9)	53 (13.8)	222 (57.8)	94 (24.5)
Purpose of medicines explained	24 (6.3)	45 (11.7)	219 (57)	96 (25)
I could ask any questions I had	10 (2.6)	77 (20.1)	230 (59.9)	67 (17.4)

### Supportive care

The mean supportive care score of the respondents was 28.84 (SD =  ± 5.78) out of 45. Most of the respondents 200 (52.1%) and 329 (85.7%) were not allowed to have companions during labor and delivery respectively. Out of the total respondents, 114 (29.7%) of them had reported that there were enough health care providers to care for them. In addition, 17 (4.4%) of mothers felt that the rooms were crowded during their stay in the facility.

Over half of the respondents, 203 (52.9%) reported that there was water in the facility and most of the participants 331 (86.2) reported the facility had electricity. Nearly half of the study participants 198 (51.8%) responded that they felt safe during their stay in the facility. Only 49 (12.8%) of mothers reported that they thought the general environment of the facility was very clean (Table 5).

**Table 5:** Distribution of supportive care items in public hospitals of Addis Ababa, Ethiopia, 2021(n = 384)

Items	No, never (%)	Yes, a few times (%)	Yes, most of the time (%)	Yes, all the time (%)
Allowed a labor companion	200 (52.1)	59 (15.4)	72 (18.7)	53 (13.8)
Allowed a delivery companion	329 (85.7)	25 (6.5)	20 (5.2)	10 (2.6)
Providers talk to me about my feeling	21 (5.5)	119 (31)	212 (55.2)	32 (8.3)
Providers supported anxieties and fears	16 (4.2)	73 (19)	255 (66.4)	40 (10.4)
Providers try to control when I have a pain	5 (1.3)	73 (19)	256 (66.7)	50 (13)
Providers paid attention when I need help	6 (1.5)	59 (15.4)	248 (64.6)	71 (18.5)
Providers took the best care for me	8 (2.1)	42 (10.9)	214 (55.7)	120 (31.3)
I trust providers regards to care	4 (1)	16 (4.2)	84 (21.9)	280 (72.9)
There was enough providers	6 (1.5)	29 (7.6)	235 (61.2)	114 (29.7)
The rooms were crowded (RC)	103 (26.8)	118 (30.8)	146 (38)	17 (4.4)
There was water in the facility	38 (9.9)	68 (17.7)	75 (19.5)	203 (52.9)
There was electricity in the facility	2 (0.5)	2 (0.5)	49 (12.8)	331 (86.2)
Feel safe in the facility	1 (0.3)	18 (4.7)	167 (43.5)	198 (51.5)
Waiting time	Very long 11 (2.9)	Somewhat long 105 (27.3)	Little long 196 (51)	Very short 72 (18.8)
The general environment of the facility	Very dirty 0	Dirty 8 (2.1)	Clean 327 (85.1)	Very clean 49 (12.8)

*RC* Reverse Coded

**Fig. 1:**
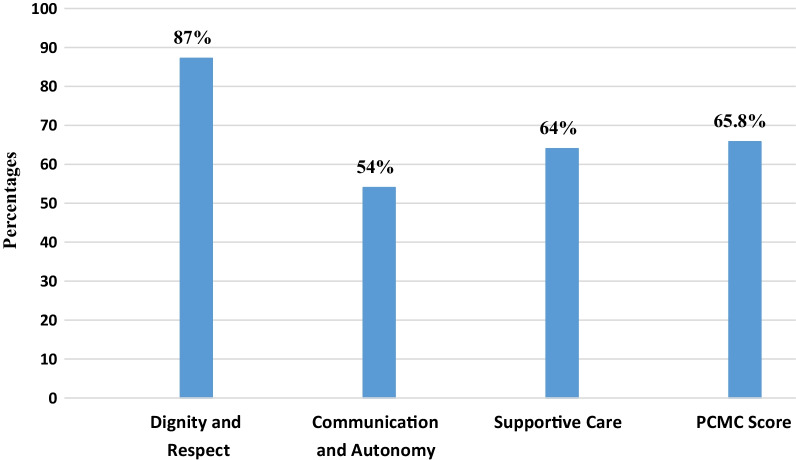
Distribution of percentage mean score of PCMC scale and subscales in public hospitals of Addis Ababa, Ethiopia, 2021

As shown in Fig. 1, the percentage mean person-centered maternity care score of the respondents was 65.8%. The percentage mean PCMC score for sub-scales was 87% for dignity and respect, 54% for communication and autonomy, and 64% for supportive care. The figure also showed that from the components of person-centered maternity care, dignity and respect recorded the highest percentage mean score followed by supportive care while communication and autonomy received the lowest mean score.

### Factors associated with person-centered maternity care

In simple linear regression residence, educational level, ANC follow-up, number of ANC, time of delivery, and complication during delivery were factors significantly associated with PCMC score. In multiple linear regression having no ANC follow up, < 4 ANC follow up, nighttime delivery, and complications during delivery were factors significantly associated with PCMC score.

By controlling the effect of all other variables in the model, mothers who have no ANC follow-up had decreased person-centered maternity care score by a factor of five times as compared to mothers having ANC follow-up (β = -5.39, 95% CI: -10.52, -0.26). Similarly, mothers having < 4 ANC visits had decreased person-centered maternity care score by a factor of four times as compared to mothers having four or more ANC visits (β = -3.99, 95% CI: -6.63, -1.36).

Respondents who gave birth at night time had decreased person-centered maternity care score by a factor of four times as compared to their counterparts (β = -3.95, 95% CI: -5.91, -1.98). Additionally, mothers who had complications during delivery had decreased PCMC score by a factor of three times as compared to mothers delivered without complications (β = -3.18, 95% CI: -6.01, -0.35) (Table 6).

**Table 6:** Bivariate and multivariate linear regression analysis for factors affecting Person-Centered Maternity Care, Addis Ababa, Ethiopia, 2021

Variables	Mean (SD)	Crude β Co-efficient (95% CI)	Adjusted β Co-efficient (95% CI)
Residence			
Urban	59.8 (10.2)	1.00	1.00
Rural	55.2 (8.5)	− 4.63(− 7.45, 1.79)**	− 1.65(− 4.82, 1.51)
Level of education			
Diploma and above	60.3 (11.2)	1.00	1.00
Unable to read and write	55.0 (9.6)	− 5.28(− 9.96, − 0.59)*	− 1.27(− 5.96, 3.42)
Primary (grade 1–8)	58.4 (9.5)	− 1.93 (− 4.64, 0.78)	− 0.50(− 3.22, 2.22)
Secondary (grade 9–12)	60.3 (10.0)	0.01(− 2.90, 2.86)	− 0.29 (− 2.48, 3.06)
ANC			
Yes	59.4 (10.0)	1.00	1.00
No	53.8 (11.0)	− 6.14(− 11.34, − 0.95)*	− 5.39(− 10.52, − 0.26)*
Number of ANC			
Four and above	60.7 (9.8)	1.00	1.00
Less than four	55.6 (9.7)	− 4.96(− 6.99, − 2.39)***	− 3.99(− 6.63, − 1.36)*
Time of delivery			
Daytime	61.1 (10.2)	1.00	1.00
Nighttime	56.8 (9.5)	− 4.3(− 6.29, − 2.31)***	− 3.95(− 5.91, − 1.98)*
Complication during delivery			
Yes	59.6 (9.6)	1.00	1.00
No	56.5 (12.2)	− 3.09(− 5.97, − 0.21)*	− 3.18(− 6.01, − 0.35)*

Keys: 1 = Reference, *CI* Confidence Interval

*p-value < 0.05, **p-value < 0.01, ***p-value < 0.001

## Discussion

Person-centered maternity care is identified as the key aspect of quality of maternity care that contributes to improved maternal and neonatal outcomes and increased institutional delivery [3].This study investigated the prevalence of person-centered maternity care and associated factors among mothers who gave birth at public hospitals in Addis Ababa city.

The study reported that the prevalence of person-centered maternity care was 65.8%. The findings of this study indicated that health care providers rarely introduced themselves, asked permission before performing examinations and procedures, involved women in decisions about their care, allowed a position of choice during delivery, explained procedures and the purpose of medications. Most of the mothers were also not allowed to have labor and delivery companions.

The finding of this study was relatively consistent with the studies done in Kenya where the proportion of person-centered maternity care was 66.9% and a community-based cross-sectional conducted study in Dessie town where the percentage mean person-centered maternity care score was 64.5% [2, 18]. This might be due to the similarity in study design and the nature of study participants.

However, the result of this study was lower than a study conducted in Ghana where more than 75% of women experienced high person-centered maternity care scores [10]. This possible reason could be due to socio-cultural and socio-economic differences of the study participants. The variation might also be due to improved quality of health care and staff training in Ghana.

On the other hand, the finding of this study was higher than a cross-sectional study done in Colombo, Sri Lanka where the mean person-centered maternity care score was 42.3 out of 90. The reason might be due to the variation in the study setting and the participant’s level of reporting the care. The results of this study also showed that a higher level of person-centered maternity care than a study conducted in Bahir Dar town where the prevalence of person-centered maternity care was 57%. The difference might be due to the study period and data collection procedure variation. In addition, it might be due to service improvement in Addis Ababa public hospitals.

The current study found that respondents who had no ANC follow-up, less than four ANC visits, nighttime delivery, and complications during delivery were factors significantly associated with person-centered maternity care.

Respondents who had no ANC follow-up had significantly decreased person-centered maternity care scores as compared to respondents who have ANC follow-up. This finding was in line with the study done in Eastern Ethiopia [23]. That was due to the fact that mothers who had no ANC might not have experience with services and they might not develop the confidence to communicate with health care providers in the facility.

Mothers who had fewer than four ANC visits had a negative association with person-centered maternity care as compared to mothers who have four or more visits. It was because incomplete ANC follow-up could make the mothers not familiar with health care providers that could affect person-centered maternity care. In addition, having infrequent ANC follow-up might decrease the utilization of maternal health services that affect the interaction of clients with health care providers and the mothers might perceive that they did not receive person-centered care.

Women who delivered at nighttime had experienced poor person-centered maternity care as compared to women who delivered during the daytime. This was consistent with a study conducted in Dessie town [2]. The possible justification could be due to the effect of work overload, physical exhaustion, and the small number of health professionals at nighttime. Hence, health care providers might not focus on PCMC. Additionally, there might be a lack of resources or weak supervision during the nighttime that could affect the mother’s experience of person-centered care.

Respondents who had complications during delivery had significantly decreased person-centered maternity care scores as compared to respondents without complications. This was consistent with a study done in Bahir Dar [22]. The reason might be mothers who had complications during delivery might perceive that they experienced complications due to the poor quality of care they received from health care providers. Moreover, mothers with a complication during childbirth care stayed in the facility for a long time without getting support from their families. Besides, they might not be satisfied with the services they received from providers.

## Strength and limitation

Strength; the study is the second of its kind in Ethiopia to assess the prevalence and factors affecting person-centered maternity care using a standard validated tool in different countries.

The limitation of this study was the cross-sectional nature of the study design does not assess the cause-effect relationship of different variables with person-centered maternity care. The other limitation of the study was conducted only in public health hospitals that might not represent the proportion of person-centered maternity care in private health institutions and government health centers. Further qualitative and quantitative studies are recommended to identify factors that affect person-centered maternity care in both governmental and private health facilities from point of life experiences.

## Conclusion

The finding of this study revealed that the proportion of person-centered maternity care during childbirth in public hospitals of Addis Ababa was 65.8%. ANC follow-up, number of ANC follow-up, time of delivery, and complications during delivery were factors significantly associated with person-centered maternity care. The factors affecting person-centered maternity care are manageable to interventions. Therefore, Policymakers should develop and implement guidelines about person-centered maternity care. Training should be given to health care providers on the importance of person-centered maternity care and patient and provider rights. Hospital managers should increase the number of staffs who got PCMC training, especially during nighttime to improve the provision of person-centered maternity care. Health care providers should implement person-centered maternity care for all mothers who gave birth in the health care facility.

## Data Availability

Both Epi-data version 4.6 and SPSS version 25 data of this article is available up on reasonable request from the authors.

## Abbreviations

ANC: Ante Natal Care
CI: Confidence Interval
LMICs: Low and Middle Income Countries
PCC: Person centered care
PCMC: Person Centered Maternity Care
WHO: World Health Organization
